# Formal Caregivers' Exposure to Workplace Violence in the Swiss Home-care Setting: A Cross-sectional Study

**DOI:** 10.1016/j.shaw.2026.07.002

**Published:** 2026-07-14

**Authors:** Baptiste Lucien, Sabine Hahn, Catherine Busnel, Sandra Zwakhalen

**Affiliations:** 1 Department of Health Services Research, Faculty of Health, Medicine and Life Sciences, Maastricht University, Maastricht, Netherlands; 2 Haute École Arc Santé, HES-SO University of Applied Sciences and Arts, Western Switzerland, Neuchâtel, Switzerland; 3 Geneva Institution for Home-care and Assistance (Imad), Geneva, Switzerland; 4 Applied Research & Development in Nursing, Division of Health Professionals, Bern University of Applied Sciences, Bern, Switzerland

**Keywords:** Health personnel, Home-care services, Workplace aggression, Workplace violence

## Abstract

**Background:**

Workplace violence against formal caregivers is a serious concern, affecting both their well-being and the quality of care. While workplace violence in hospitals is well studied, research on violence in home-care settings remains scarce. This study examines the prevalence, frequency, types, consequences, and risk factors of workplace violence experienced by formal caregivers from care receivers in home-care.

**Methods:**

A descriptive cross-sectional study was conducted in 2022 in a home-care organisation in French-speaking Switzerland using an electronic survey. Formal caregivers were selected using convenience sampling. Data were analysed using descriptive statistics, and Pearson's chi-square and Fisher's exact tests. The Holm-Bonferroni method was applied for the post-hoc analysis.

**Results:**

Among 200 participants, 42% reported experiencing at least one form of workplace violence in the past year. Following a workplace violence event, nine affected participants took medical leave, with four being absent for more than seven days. Nursing assistants were less likely to experience workplace violence than registered nurses and health specialists. Formal caregivers with 1-4 years of experience were at higher risk of workplace violence compared to those with longer professional experience.

**Conclusion:**

This study is one of the first to examine workplace violence towards formal caregivers in the European home-care context, revealing concerning rates of workplace violence with significant consequences for caregivers, and highlighting the urgent need for greater attention to this phenomenon. Addressing workplace violence in home-care is essential to safeguarding caregiver well-being and preventing workforce shortages.

## 1 Introduction

Workplace violence (WPV) against formal caregivers such as nurses and physicians, perpetrated by care recipients (commonly referred to as patients or clients), has become a critical concern globally [1]. According to the World Health Organisation (WHO), WPV is defined as ‘incidents where employees are abused, threatened, assaulted or subjected to other offensive behaviour in circumstances related to their work’ [2]. It can manifest in various forms, including physical violence (e.g., hitting, punching), non-physical violence (e.g., threats, stalking), sexual aggression (e.g., rape, forced kissing/touching), and sexual harassment (e.g., sexism, sexual compliments) [3].

WPV poses significant risks to the safety and well-being of formal caregivers while also adversely affecting the quality of care they provide. [1,3,4]. The consequences of WPV can include injuries such as fractures or chronic pain, and in rare cases, even death [5]. WPV can also lead to psychological consequences such as post-traumatic stress disorder, decreased job satisfaction, feelings of insecurity, burn-out syndrome, and depression [3,4,[6], [7], [8]]. Additionally, in terms of its impact on formal caregivers' security, WPV results in an increase in the shortage of healthcare workers identified by the WHO, posing a significant concern for the future sustainability of healthcare [9].

Despite the growing concerns surrounding WPV, its prevalence remains alarmingly high [1,3,10,11]. Yusoff et al. [1] reported a 12-month prevalence rate of WPV towards formal caregivers ranging from 45.6% to 90%, depending on the type of WPV (physical or non-physical), country, and healthcare setting. Specifically, 66.2% of formal caregivers in hospital settings and 67.1% in psychiatric care settings reported exposure to WPV. While this phenomenon has been extensively studied in hospital, psychiatric, and nursing home environments, it has been largely understudied in the context of home-care [1,3].

As the population ages and the prevalence of chronic conditions increases, the demand for home-care settings is increasing, presenting both unique challenges and opportunities for the delivery of personalised, continuous care in a familiar environment [12]. In this context, preventing WPV is critical for the provision of quality home-care and optimising formal caregivers' work performance [13]. The home-care setting presents distinct challenges regarding WPV, primarily given that formal caregivers often work alone and face WPV from various sources, including care receivers as well as their relatives and their environment (e.g., dangerous neighbourhoods and dog bites) [3].

In the home-care setting, the literature indicates that up to 61.3% of formal caregivers experience some form of WPV annually [3]. Furthermore, depending on the area, up to 27.5% of these caregivers reported experiencing physical violence annually [3]. High incidences of sexual harassment have also been documented; according to Hanson et al. [14], 27.6% of formal caregivers experienced sexual harassment and 12.8% experienced sexual aggression within the last year of caregiving in the home-care setting. Despite this global prevalence, research on WPV in the home-care sector is predominantly available from the USA [3]. One of the few studies conducted in Europe found that in 2019, 54.7% of formal caregivers in Switzerland experienced WPV from care receivers with dementia living at home [15]. Of these incidents, 51% involved non-physical violence, and 14.8% included physical violence [15]. However, this study appears to be the only study outside of the USA focusing on WPV in the home-care setting, but with a specific focus on care receivers with dementia—a recognised risk factor for such violence [3].

Research from the USA may provide limited insight into the situation due to its specificities in healthcare systems (e.g. private healthcare system), cultural and societal contexts (e.g. right to bear arms), and working conditions, compared to those from Europe. While WPV exists in home-care settings in both countries, variations in healthcare structures, workforce conditions, cultural norms, policies, and training approaches may yield differing experiences and reporting patterns. Therefore, the present study aims to examine the prevalence, frequency, type, consequences, and determinants of WPV experienced by formal caregivers in the home-care setting in Switzerland.

## 2 Material and method

### 2.1 Study design

We employed a cross-sectional study design to determine the prevalence, frequency, type, consequences, as well as the determinants of WPV experienced by formal caregivers in the home-care setting in Switzerland. The STRengthening the reporting of OBservational studies in Epidemiology (STROBE) checklist was followed for the reporting of this study [16].

### 2.2 Setting and context

This study was conducted in one public home-care organisation operating in the French-speaking region of Switzerland. This organisation is divided into 5 agencies composed of 2,406 professionals. These professionals collectively provided care to 18,556 care receivers living at home, reflecting the high demand for home-based healthcare services in the region. The agencies vary in size, with some serving predominantly urban areas and others semi-urban communities, ensuring coverage across diverse care contexts.

### 2.3 Participants and sampling

The eligible study population consisted of all formal caregivers of one home-care organisation. A total of 686 formal caregivers, including nurses (with a 3-year Bachelor's degree in Nursing), health care specialists (with a 3-year apprenticeship concentrating on fundamental care) and nursing assistants (with a 2-year education focusing on providing basic care under nurses' supervision), who were employed in the home-care organisation, were eligible. Participants had to meet the following inclusion criteria: (1) be aged 18 years or older, (2) be directly involved in providing home-care to care receivers living at home, and (3) have sufficient proficiency in French to read and complete the questionnaire. Eligibility was assessed at the beginning of the survey via screening questions in the first section of the questionnaire.

### 2.4 Measures

In the present study, the French version of the Survey of Violence Experienced by Staff of the home-care setting (SOVES-Fr-HC) was used. Based on the SOVES-G-R, the SOVES-Fr-HC is a six-section questionnaire consisting of 70 items in total and requiring between five and 25 minutes to complete [17]. Based on the aim of the present study, sections A–D of the SOVES-Fr-HC are included in this study to focus only on prevalence, type, consequences and determinants of WPV towards formal caregivers in the home-care setting.

• Section A inventories formal caregivers' socio-demographic and background characteristics (e.g. gender, age, profession, work experience in healthcare setting, work experience in present position, level of employment, and the time spent in direct care-receiver contact).
• Section B explores the participants' exposition of WPV across their entire career in healthcare settings with dichotomous and multiple-choice questions addressing the professional life exposure to physical WPV, non-physical WPV, sexual harassment, and sexual aggression.
• Section C comprises multiple-choice questions that investigate the sources (e.g. care receivers, relatives or others), prevalence, and frequency of exposure to various types of WPV in the past 12 months within the participants' current workplace.
• Section D examines the consequences of violence through dichotomous and multiple-choice questions regarding the effects of the most recent WPV incident experienced by the caregiver, such as taking sick leave or experiencing emotional consequences.

Before the start of the current study, the authors developed and validated a French adaptation of the SOVES-G-R specifically tailored for the home care setting, referred to as SOVES-Fr-HC. The translation and cultural adaptation followed established cross-cultural procedures including forward and backward translation and expert panel review. Construct validity and reliability of the POAS-S and POIS subscales were assessed in the methodological validation study. Importantly, the present study used only Sections A–D of the SOVES-Fr-HC, which consist of categorical variables assessing exposure, prevalence and consequences of workplace violence rather than psychometric scales [18].

### 2.5 Data collection

Data were collected between March and November 2022 using a convenience sampling method. The LimeSurvey Software, Version 3.20.1 [19], was utilised to develop an electronic version of the SOVES-Fr-HC questionnaire. Upon obtaining consent from the management of the home-care organisation for participation in the study, a survey URL was generated. The managers of the five home-care agencies distributed the URL, along with an information letter, a video presentation of the research project, and a detailed project description, to all formal caregivers. Additionally, the first author conducted two face-to-face presentations of the research project in March aiming to expand recruitment avenues. Participants accessed the survey by clicking on the provided URL, where they were first required to provide informed consent. The initial page of the survey consisted of an electronic version of Section A of the SOVES-Fr-HC. Participants who answered that they did not meet the inclusion criteria, as determined by their responses to the socio-demographic questions in Section A, were unable to proceed with the survey. To maximise participation, reminder emails were sent by the managers of the five home-care agencies at 8, 16, and 24 weeks.

### 2.6 Data analysis

Data were analysed using R software V.4.1.2 with dplyr, vcd, fmsb packages [20]. Socio-demographic and background characteristics were analysed using descriptive statistics (frequencies). Relationships between WPV and potential determinants (e.g., gender, age, profession) were assessed using Pearson's chi-square test when its assumptions were met, and Fisher's exact test otherwise. To account for the risk of type I errors due to multiple testing, post-hoc pairwise comparisons were performed using the Holm-Bonferroni correction method [21]. For all statistical tests conducted, a two-sided *p*-value of less than .05 was considered statistically significant. As the primary aim of this study was descriptive and exploratory, the statistical analysis focused on descriptive statistics and bivariate associations rather than multivariable modelling.

### 2.7 Ethical considerations

This study was conducted in accordance with the ethical principles outlined in the Declaration of Helsinki. The local Swiss ethics board confirmed that the study did not warrant a full ethics application and did not fall under the Swiss Federal Act on research involving human beings (REQ-2021-01172). All the data were collected anonymously with LimeSurvey after each participant consented [19]. Participants had the option to withdraw from the study at any time. If a participant exited the survey mid-way, all previously provided responses were excluded from the study. The survey was hosted on a secure server managed by the University of Applied Sciences and Arts, Western Switzerland (HES⁠-⁠SO), ensuring that all data were stored and processed in accordance with Swiss data protection regulations and institutional ethical standards. No data were stored on external or commercial servers, and access to the data was restricted to the research team.

## 3 Results

The survey URL was distributed to 686 formal caregivers. Of these, 202 accessed and completed the online survey (response rate: 29.2%). Further details on the characteristics of the participants are presented in Fig. 1 and Table 1.

**Fig. 1:**
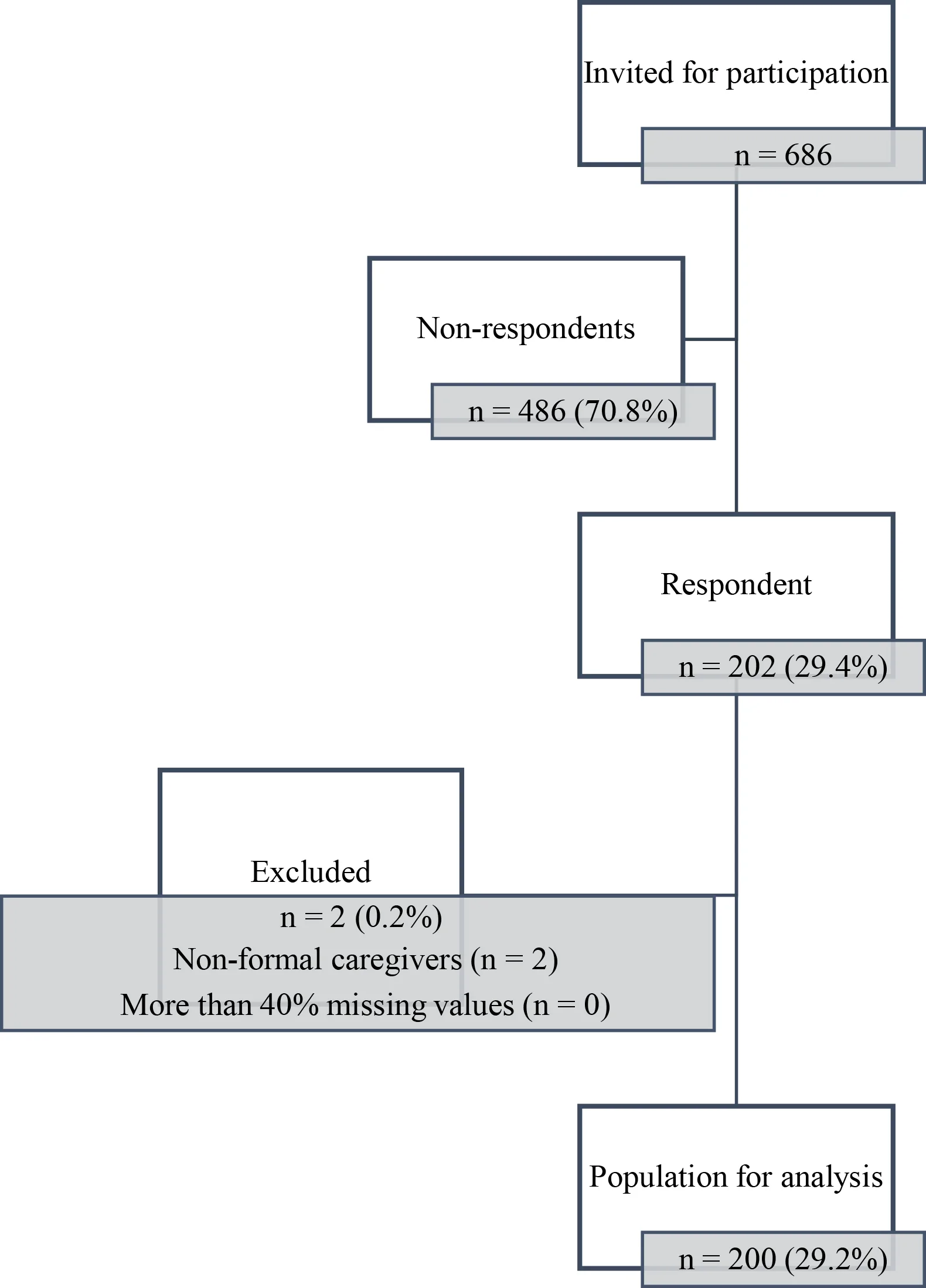
Flow-chart of the study participants.

**Table 1:** Characteristics of the participants and determinants of workplace violence (WPV)

Characteristics	Total (*n* = 200)	Exposure to WPV in the previous 12 months (total *n* = 84, 42%)		
		*n* (%†)	χ^2^(d.f.)	*p*-value
**Gender**				
Female	163	69 (42.3)		
Male	37	15 (40.5)	0.04 (1)	0.84
**Age (years)**				
18 to 29	21	11 (52.4)		
30 to 45	101	44 (43.6)		
46 to 55	59	23 (39.0)		
More than 55	19	6 (31.6)	2.10 (3)	0.55
**Profession**				
Registered nurses	113	51 (45.1)		
Health specialists	47	25 (53.2)		
Nursing assistants	40	8 (20.0)	**10.82 (2)**	**0.00**∗
**Work experience in healthcare setting (years)**				
0 to 4	13	5 (38.5)		
5 to 10	33	17 (51.5)		
11 to 15	53	23 (43.4)		
More than 15	101	39 (38.6)	1.81 (3)	0.61
**Work experience in the current designation (years)**				
Up to 1	20	2 (10.0)		
1 to 4	67	34 (50.7)		
5 to 10	53	27 (50.9)		
More than 10	60	21 (35.0)	**13.46 (3)**	**0.00**∗
**Level of employment**				
Up to 50%	6	0		
51 to 89%	121	57 (47.1)		
90 to 100%	73	27 (37.0)	**6.39 (2)**	**0.04**∗
**Time spent in direct care receivers' contact**				
Less than 30%	29	5 (17.2)		
30 to 60%	55	18 (32.7)		
More than 60%	116	61 (52.6)	**14.58 (2)**	**0.00**∗

Note: χ2, chi-square; df, degrees of freedom.

Bold values indicate statistically significant results (*p* < 0.05).

∗ All chi-squares were statistically significant at *p* < 0.05.

† Percentages refer to the proportion of participants exposed to WPV within each characteristic category.

### 3.1 12-months prevalence, frequency, consequences and source of workplace violence towards formal caregivers

In this study, 42% (*n* = 84) of the formal caregivers reported experiencing at least one instance of WPV from a care receiver residing at home or a relative during the 12 months preceding data collection. Among all the participants, 14% (*n* = 29) reported having experienced at least one instance of physical violence, 39% (*n* = 78) non-physical violence, 8% (*n* = 16) sexual harassment, and 2.5% (*n* = 5) sexual aggression. In the past 12 months, 53 of the participants (26.5%) reported experiencing more than two incidents of non-physical workplace violence, primarily perpetrated by care receivers living at home.

Of the 29 participants who experienced physical violence, seven participants were injured during the last violent event. Two of them suffered severe injuries necessitating medical follow-up and/or treatment. Among the participants experiencing WPV in the past 12 months, nine took medical leave as a result, with four being absent for a period exceeding seven days. Among participants who experienced WPV in the past 12 months, 49 out of 75 reported feeling moderately to extremely stressed by the most recent act of non-physical WPV, and 14 out of 28 reported feeling moderately to extremely stressed by the most recent act of physical WPV.

### 3.2 Determinants of workplace violence

The results of Pearson's Chi-Square tests demonstrated that the 12-month prevalence of WPV was significantly associated with participants' profession (χ^2^ = 10.82, d.f. = 2, *p* < .001), professional experience in the current position (χ^2^ = 13.46, d.f. = 3, *p* < .001), level of employment (χ^2^ = 6.39, d.f. = 2, *p* = .04), and time spent in direct contact with care receivers living at home (χ^2^ = 14.58, d.f. = 2, *p* < .001) (Table 1).

Post-hoc Holm-Bonferroni analyses revealed that nursing assistants were significantly less likely to experience WPV compared to registered nurses (*p* = .009) and health specialists (*p* = .051, trend level). Regarding work experience in the current position, formal caregivers with less than one year of experience were less likely to experience WPV than those with 1–4 years (*p* = .007) and 5–10 years (*p* = .007), while other pairwise comparisons were not significant. For level of employment, participants employed up to 50% were less likely to experience WPV than those employed 51–89% (*p* = .002) and 90–100% (*p* = .002), with no significant difference between 51–89% and 90–100%. Finally, regarding time spent in direct care, caregivers spending more than 60% of their time in direct contact were more likely to experience WPV than those spending 30–60% (*p* = .031) or less than 30% (*p* = .002) of their time.

## 4 Discussion

The results of the present study show that more than one out of three of the formal caregivers had experienced at least one event of WPV during the past 12 months. Registered nurses and health specialists are significantly more likely to experience WPV than nursing assistants. In addition, formal caregivers with 1–10 years of experience in their current position were more likely to experience WPV than their colleagues. Workplace violence against formal caregivers is a global concern, yet research specifically addressing WPV in European home care settings remains notably scarce and underexplored [3,4]. International studies often originate from North America and Asia, leaving significant gaps in the understanding of WPV within the unique context of European home care [4]. While general European estimates suggest a 12-month prevalence of non-physical WPV at approximately 36.6% and physical WPV at 20.1% across all healthcare sectors, and some studies among nurses in European countries report non-physical violence affecting 54% and physical violence 20%, detailed data for home care is particularly limited [22,23]. These findings indicate that while the rate of physical WPV aligns with the findings reported in the study conducted in Switzerland by Schnelli et al. [24], the prevalence of non-physical WPV is lower in the present study. These variations in rates of physical and non-physical WPV can be attributed to differences in sample characteristics and the specific context of the studies. Schnelli et al. (2021) focused on WPV experienced by formal caregivers specifically dealing with older adults diagnosed with Alzheimer's disease [24]. The literature has demonstrated that Alzheimer's disease is likely to increase the risk of WPV occurrence [3,25]. Even though 42% of the participants of the present study had experienced WPV in the 12-months preceding the study, the rate of WPV in the Swiss home-care setting appears to be lower than that in other settings and other countries [11]. This aligns with findings from broader meta-analyses indicating that the overall prevalence of WPV against healthcare workers has been observed to be lower in European countries (20.1%) compared to North America, Asia, and Australasia [11]. The present study thus offers a crucial contribution by providing specific prevalence data and insights into the characteristics of WPV within a European home care context, an area previously characterised by limited evidence and underreporting, thereby addressing a significant gap in the literature. Regarding sexual forms of violence, 8% of the participants reported experiencing sexual harassment and 2.5% reported exposure to sexual aggression in the home-care setting, highlighting the presence—albeit at different levels—of both types of sexual violence in this context. These prevalence rates are notably lower than those reported in previous studies conducted in the USA home-care setting [14,26]. Several factors could contribute to this discrepancy, including differences in healthcare systems, legal frameworks, cultural norms, and reporting behaviours. In addition, underreporting cannot be excluded, particularly for sexual violence, which may be less frequently disclosed because of stigma, embarrassment, fear of trivialisation, or uncertainty about what should be considered workplace violence in the home-care context. Qualitative studies would be useful to better understand how formal caregivers interpret, disclose, and report sexual violence in this setting.

Regarding the consequences of WPV, participants who experienced WPV in the present study reported experiencing minimal to moderate levels of stress. This finding should be interpreted with caution, as stress was assessed using a single-item measure referring to the most recent workplace violence incident and may therefore underestimate the broader psychological impact of repeated or more severe events. However, this is surprising given the documented psychological consequences, such as post-traumatic stress syndrome and sleep problems, highlighted in other studies [1]. One plausible explanation is that respondents reported their most recent incident, rather than the most severe incident experienced during the past 12 months. In addition, this difference may be related to formal caregivers' perceptions of workplace violence and their attitudinal responses. Previous research shows that formal caregivers exposed to workplace violence may experience feelings of humiliation and self-blame and may rely on coping strategies such as denial, potentially leading to an underestimation of perceived stress [27]. Previous research in psychiatric and emergency settings has revealed that a substantial proportion of formal caregivers either regard WPV as an inherent aspect of their work environment or fail to recognise its occurrence altogether [3,[27], [28], [29]]. This lack of perception of WPV hinders the implementation of strategies to manage and prevent WPV incidents, thereby increasing the risk of recurrent WPV incidents among formal caregivers. However, increased incidents of WPV towards formal caregivers result in higher rates of medical leave, exacerbating the shortage of available caregivers. This shortage, in turn, compromises the quality of care provided to care receivers [9].

The results of the present study showed that formal caregivers with less than one year of work experience in the home-care setting are less likely to experience WPV than their peers with 1 to 10 years of experience. These findings contrast with existing literature, which suggests that formal caregivers with limited experience in the home-care setting face a higher risk of workplace violence compared to their more experienced counterparts [1,3]. Few studies have comprehensively addressed the influence of job position, time dedicated to direct care for care receivers, and the formal caregivers' years of experience in their current positions as factors contributing to WPV in the home-care setting [30,31]. This fact could be explained by the lack of awareness of what is defined as WPV in this setting; formal caregivers with less than 1 year of experience are less familiar in identifying and reporting a WPV event [3]. According to recent evidence, the home-care setting presents specific types of WPV and formal caregivers with minimal experience in this setting minimise the incident and refrain from reporting it [3,27]. In addition, inexperienced formal caregivers are often supported by an experienced counterpart at the beginning of their career, reducing the likelihood of being left alone without support in the care receiver's home [32].

### 4.1 Strength and limitation

This study is one of the early investigations of specific types of WPV, such as sexual aggression and harassment, experienced by formal caregivers in the home-care setting within Europe. Furthermore, it contributes to the limited body of research exploring WPV towards formal caregivers, specifically in the home-care settings of French-speaking regions in Switzerland. Despite these strengths, several limitations should be noted.

First, the use of a convenience sampling strategy (29.2% response rate) may have introduced selection bias, as voluntary participation could result in the over- or underrepresentation of individuals with particular interest, expertise, or personal experience related to WPV. In addition, the respondents included a higher proportion of registered nurses (56.5%) compared to other professional categories, which may not fully reflect the distribution of the organisation's workforce and could affect the generalizability of the findings. Future studies should compare respondent demographics to organisational workforce data to assess representativeness. In addition, the survey did not collect information on formal caregivers' training history regarding workplace violence prevention, which could represent an important explanatory factor for differences in exposure or reporting of WPV. Second, the study design did not prevent the possibility of duplicate responses, as participants could theoretically have completed the survey more than once using different devices or browsers. While the risk is considered low given the professional context and absence of incentives, this remains a potential source of bias. Third, the definition of WPV used in this study assumes an intentional act to physically or psychologically harm formal caregivers. In practice, several care receivers involved in WPV may not intend to cause harm, even if their behaviour results in injury or psychological distress. This definitional constraint may lead to underreporting or misclassification of incidents, particularly those perceived as unintentional or related to cognitive impairments (e.g., dementia). In addition, the survey did not collect detailed information on care-receiver clinical characteristics, including dementia, which limited the possibility of stratified analyses according to care-receiver conditions. Consequently, the exclusion of dementia-specific WPV limits comparability to other Swiss data and may underestimate the prevalence and complexity of WPV in home care. Stratified analyses by care-receiver conditions could be considered in future research. Finally, while this study provides valuable insights, its cross-sectional design limits the ability to infer causal relationships—particularly between WPV and potential determinants (e.g., age, professional experience, work setting) or consequences (e.g., psychological distress, job satisfaction). Future longitudinal research is warranted to better understand these relationships and monitor trends in WPV over time in the home-care setting.

### 4.2 Implications for practice

In the Swiss home-care context, formal caregivers frequently provide care alone in care-receivers’ homes without immediate access to colleagues or institutional security measures. This organisational characteristic may increase formal caregivers' perceived vulnerability when facing aggressive behaviours. This study underscores the diversity and complexity of WPV in the home-care setting. Beyond the more commonly addressed forms of physical and verbal aggression, a notable proportion of formal caregivers also reported experiencing sexual harassment (8%) and, to a lesser extent, sexual aggression (2.5%). These findings highlight the need for a clear and shared understanding of workplace violence among formal caregivers, ensuring that all its forms are consistently recognised and addressed within care organisations. Home-care organisations should implement comprehensive training programmes to help formal caregivers recognise all forms of WPV and require systematic reporting. Such training should be tailored—when possible—to account for the participants' profession and professional experience in the current position, particularly in light of findings suggesting that nurses and less experienced formal caregivers may be more vulnerable to WPV. Additionally, organisations should assess the specific needs of formal caregivers regarding workplace violence prevention, intervention, and postvention. This includes providing targeted strategies to mitigate risks, equipping caregivers with de-escalation techniques, and ensuring access to psychological and legal support following WPV incidents. Although participants in this study reported minimal to moderate stress, evidence from the literature indicates that exposure to workplace violence can have serious psychological consequences [1,33,34]. Therefore, a proactive and multifaceted approach—including mental health support—is essential for safeguarding formal caregivers’ safety and well-being in the home-care setting. Furthermore, given the significant consequences of WPV on formal caregiver well-being and job retention, home-care organisations must establish clear policies and procedures to address WPV incidents. Security measures, such as emergency devices, should be provided to formal caregivers, and risk assessments should be conducted to avoid sending formal caregivers unescorted to potentially dangerous environments. Finally, considering the emotional and professional toll that WPV can take on formal caregivers, organisations should ensure access to psychological support services and establish structured follow-up procedures after an incident. These measures are not only essential to staff well-being and retention but also contribute to the continuity and quality of care delivered to care receivers.

### 4.3 Implications for research

Exploring WPV within home-care settings remains an urgent priority, offering an opportunity to deepen understanding of its prevalence, characteristics, and impact on formal caregivers. Specific forms of violence, particularly sexual harassment and aggression, are underexplored and warrant focused investigation. Future research should employ large, representative datasets encompassing diverse forms of violence and include relevant covariates, such as training history or awareness of WPV prevention, to better interpret differences across experience levels. Quantitative approaches are essential for accurately estimating the prevalence of sexual aggression and harassment, while complementary qualitative studies, including interviews with managers, could contextualise findings within the Swiss home-care infrastructure and provide insights into potential underreporting due to stigma, fear of repercussions, or organisational and cultural norms. Further research should examine the characteristics of care receivers, formal caregivers' perceptions of WPV, and the effectiveness of interventions aimed at reducing both prevalence and impact of violence. Interventions must be tailored to the home-care setting, as many existing strategies (e.g., team-based approaches or seclusion) may be impractical or infringe upon care receivers' rights. Finally, multivariate statistical analyses, such as logistic regression adjusting for confounders (e.g., gender, age, professional category), are recommended to identify independent risk factors and inform the development of evidence-based prevention and mitigation strategies.

## 5 Conclusion

This study contributes to the limited body of evidence examining workplace violence towards formal caregivers in the European home-care setting. The findings highlight that WPV has tangible consequences, including medical leave, underscoring the need for interventions and support to protect formal caregivers and address the global workforce shortage identified by the WHO. Significant associations were found between WPV and factors such as profession, work experience, level of employment, and time spent in direct contact with care receivers, indicating that preventive measures should consider these determinants. To enhance the practical utility of these findings, home-care organisations should implement targeted interventions such as WPV awareness and prevention training, clear reporting systems, and organisational policies to support formal caregivers following violent incidents. Addressing WPV in this manner can improve the safety and well-being of formal caregivers, reduce compromised care for care receivers, and ultimately strengthen the quality and sustainability of home-care services. This study underscores the urgent need to prioritise WPV mitigation in home care through concrete, actionable strategies tailored to this unique setting.

## CRediT authorship contribution statement

**Baptiste Lucien:** Writing – review & editing, Writing – original draft, Visualization, Validation, Resources, Project administration, Methodology, Investigation, Formal analysis, Data curation, Conceptualization. **Sabine Hahn:** Writing – review & editing, Supervision. **Catherine Busnel:** Investigation, Data curation. **Sandra Zwakhalen:** Writing – review & editing, Supervision.

## Statement on the use of AI tools

The authors declare that artificial intelligence (AI) tools were not used to generate, analyse or interpret the results presented in this manuscript.

Language editing support using OpenAI's ChatGPT (version GPT-4, OpenAI, 2025) was employed under the supervision of the corresponding author, solely for the purpose of enhancing clarity and fluency in the English language.

## Conflicts of interest

The authors declare no competing interests.
